# Mobile health applications for the management of primary hypertension: A multicenter, randomized, controlled trial

**DOI:** 10.1097/MD.0000000000019715

**Published:** 2020-04-17

**Authors:** Ke Gong, Yu-Ling Yan, Yu Li, Jun Du, Jing Wang, Yue Han, Ya Zou, Xin-yu Zou, Hong Huang, Qiang She

**Affiliations:** Department of Cardiology, The Second Affiliated Hospital of Chongqing Medical University, Chongqing, China.

**Keywords:** management, mobile health applications, primary hypertension, randomized

## Abstract

**Background:**

Hypertension is 1 of the major global public health challenges, which means that patients with hypertension need more measures to control their blood pressure. Currently, smart phones and applications are developing rapidly, and mobile health applications are used to manage hypertension, but evidences related to effectiveness are limited.

**Objective:**

The purpose was to assess the impact of m-Health apps on blood pressure control, medication adherence.

**Methods:**

480 participants were randomly assigned to the intervention and control groups. The intervention group used the “Yan Fu” app to manage their blood pressure, and the control group did not use any m-Health apps. The outcomes were changes in blood pressure, the percentage of participants with their blood pressure under control and medication adherence.

**Results:**

At the end of the study, the baseline characteristics between the 2 groups had no statistically differences (*P* > .05). Participants in the 2 groups all had lower systolic blood pressure and diastolic blood pressure than they did at baseline, and the intervention group demonstrated a significantly greater systolic blood pressure and diastolic blood pressure reduction than the control group (*P* < .05). Additionally, the percentage of participants with controlled blood pressure was higher in the intervention group (*P* < .05). The medication adherence of the intervention group was much higher than that of the control group (*P* < .05).

**Conclusion:**

M-Health apps are effective for hypertension management, it can favor the medication adherence and blood pressure control. Perhaps m-Health apps can be promoted in the blood pressure control.

Trial Registration: This study was registered in the Chinese Clinical Trial Registry under the number ChiCTR-IOR-17012069.

## 1 Introduction

Hypertension is a leading risk factor for cardiovascular and cerebrovascular diseases.^[1]^ In China, there is a high prevalence of hypertension with low rates of disease awareness, treatment and blood pressure control. Many patients don’t control their blood pressure well because of poor medication adherence, therapeutic inertia and lack of patient engagement.Unfortunately, traditional hypertension management measurements are less efficient because of the barriers of communication between physicians and patients and a lack of detailed management.^[2]^ The Healthy China 2020 Strategic Research Report indicates that we need to transform our medical model. Mobile-Health is a new health mode recently. It can overcome the disadvantages of the traditional medical mode. Nowadays many applications based on mobile health are applied to the hypertension management. The functions of these apps are hypertension education, monitoring blood pressure and tools to promote medication adherence.^[3]^ However, the effectiveness of mobile health applications is not clear. We need to explore the effectiveness of mobile health applications on hypertension management.

## 2 Design /methods

### 2.1 Study design

This study was a multicenter, randomized, controlled trial. This clinical trial was approved by the Ethics Committee of the Second Affiliated Hospital of Chongqing Medical University. This clinical trial was registered in the Chinese Clinical Trial Registry under the number ChiCTR-IOR-17012069. Informed consent was obtained from each patient and the study protocol conforms to the ethical guidelines of the 1975 Declaration of Helsinki as reflected in a priori approval by the institution's human research committee.

**Figure 1:**
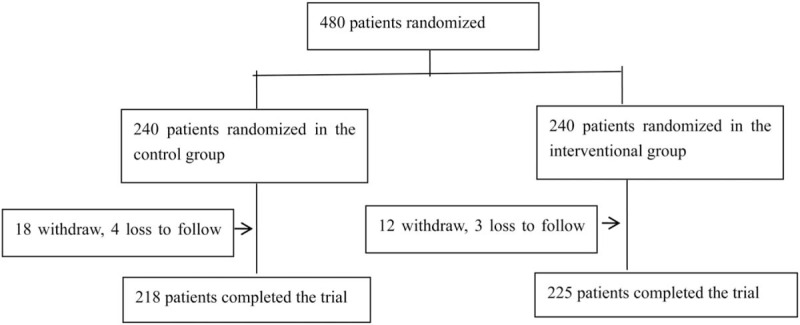
Flow chart of patients enrolled in the study.

The trial was carried out in 38 hospitals in Chongqing. All the investigators in charge of the study underwent standard training. Patients aged 18 to 79 diagnosed with primary hypertension according to the diagnostic criteria in the 2010 Chinese Guidelines for Hypertension Prevention and Treatment were recruited. A detailed list of the inclusion and exclusion criteria is shown in Table 1. Enrolled patients were randomly assigned to the intervention group and the control group in a ratio of 1:1 by using SPSS24.0 (Fig. 1). A baseline survey consisting of questions about their demographics, cardiovascular comorbidities, use of cigarettes, number of antihypertensive medication and family history was completed at the start of the trial.

**Table 1:**
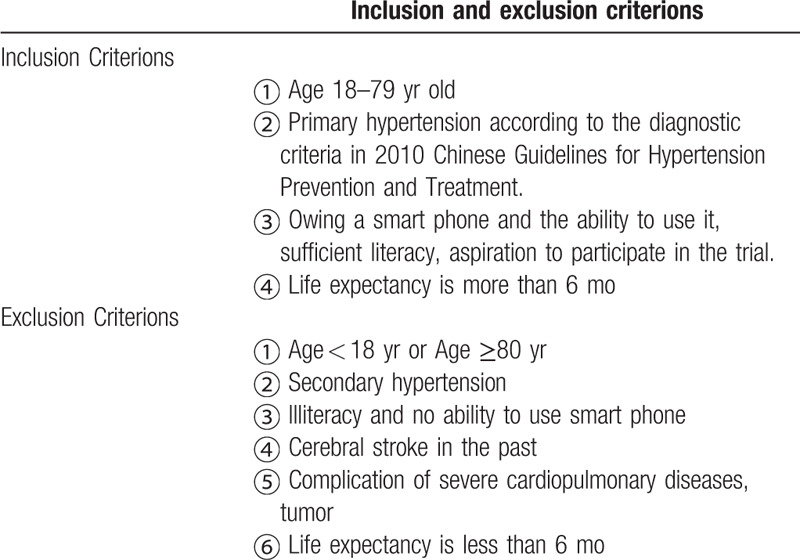
Inclusion and exclusion criterions of the trial.

### 2.2 Interventions

The participants in the intervention group downloaded the “Yan Fu” app and registered with their real name. The basic functions of the app are shown in Table 2. The investigators taught each individual how to operate the app, and helped to set medication and blood pressure monitoring schedules. Then, the app would remind the participants about the dose and timing of their drugs and blood pressure measurements. The participants were provided scientific information and suggestions about hypertension. The participants were instructed to upload their blood pressure at least once daily and record if they had taken medicine in the app. They uploaded their blood pressure data through Bluetooth between the app and the automatic sphygmomanometer, which was provided by the Shanghai O2O Care Company. If the participants did not upload the blood pressure measurements or medication record, the app would send messages to remind them. If the blood pressure was abnormal, the app would warn participants to measure their blood pressure again after taking a rest and remind them it is time to see the doctor. Patients in the control group did not use any hypertension management applications but they needed to measure their blood pressure using the same sphygmomanometer and record their blood pressure on paper every day. The participants were followed up every month to record their survival condition and blood pressure. All participants were followed up for 6 months.

**Table 2:**
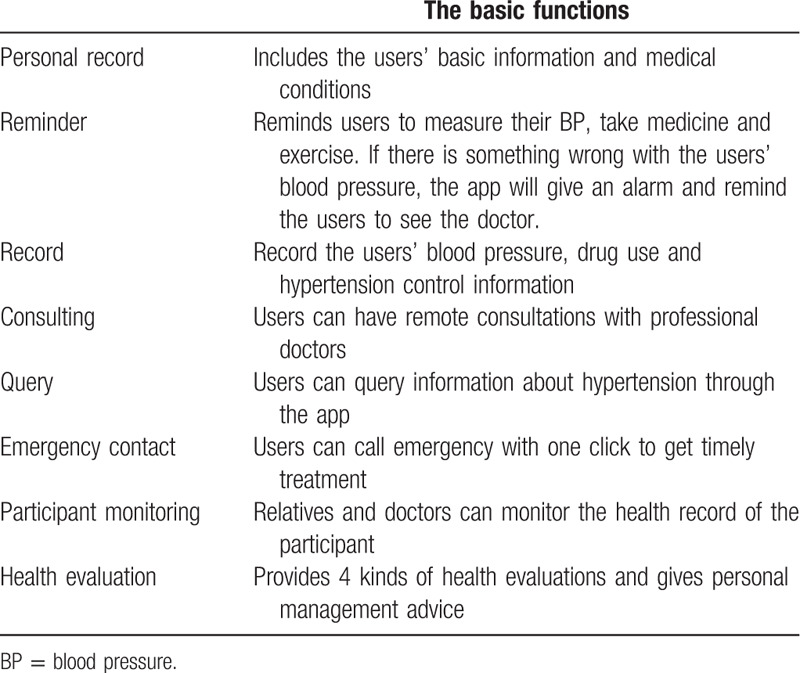
The basic functions of the app.

### 2.3 Outcomes

#### 2.3.1 Primary outcomes

The primary outcomes of this clinical trial were systolic blood pressure (SBP) and diastolic blood pressure (DBP) changes in all participants. The mean SBP and DBP at the baseline and end of this trial were calculated from the first and last 3 days’ BP respectively. The other outcome was the change in percentage of participants in the 2 groups with controlled blood pressure. According to the 2010 Guidelines for Hypertension Prevention and Treatment in China, hypertensive patients with chronic kidney disease or diabetes should have a blood pressure under 130/80. Patients with hypertension who are aged 65 years or older should have a target blood pressure of less than 150/90, and other general patients should have a target blood pressure of less than 140/90. Only both the diastolic and SBP reach the set standards, the blood pressure is categorized as controlled.

#### 2.3.2 Secondary outcomes

The Modified Morisky Scale 8 (MMS-8), which was comprised of 8 questions, was used to measure medication adherence.^[4]^ MMS-8 score less than 6 was categorized as low adherence; a score of 6–8 was categorized as medium adherence, and a score of 8 was categorized as high adherence. The details of the MMS-8 scores are shown in Table 3.

**Table 3:**
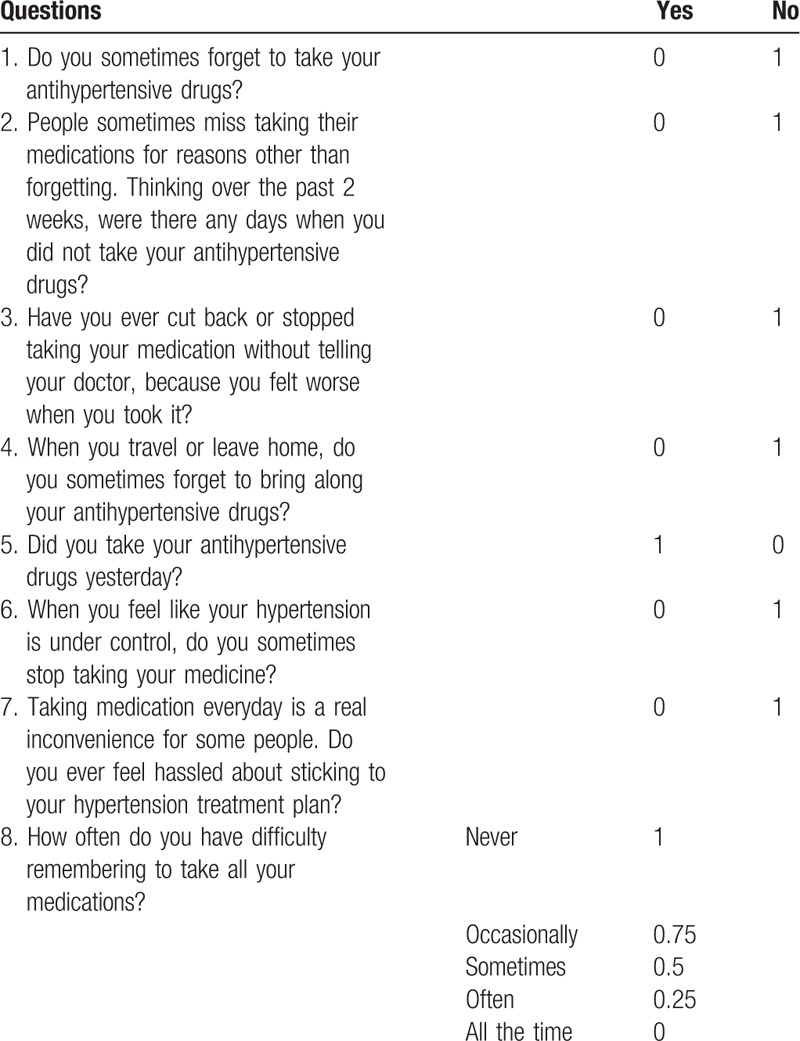
The modified Morisky scale.

### 2.4 Statistical analyses

SPSS 24.0 statistical software was used for data analysis. Quantitative datas fitted normal distribution were described using the mean and standard deviation, and Student *t* tests were used to compare the 2 samples. The qualitative findings of the study are described using frequency and percentage frequency. Chi-squared test were used to compare the qualitative datas of the trial between the 2 groups. The MMS-8 scores for medication adherence were measured via a nonparametric Wilcoxon signed-rank test. *P* < .05 was considered to be statistically significant.

## 3 Results

### 3.1 Patient characteristics

From November 2017 to November 2018,480 participants were enrolled. At last,443 participants completed trial, and 37 participants lost to follow up, of which 7 were out of contact and 30 withdrew from our study. There were 218 participants in the control group (Group A) and 225 participants in the intervention group (Group B) The baseline characteristics of the 2 groups are shown in Table 4. No significant differences were observed between both groups in baseline datas.

**Table 4:**
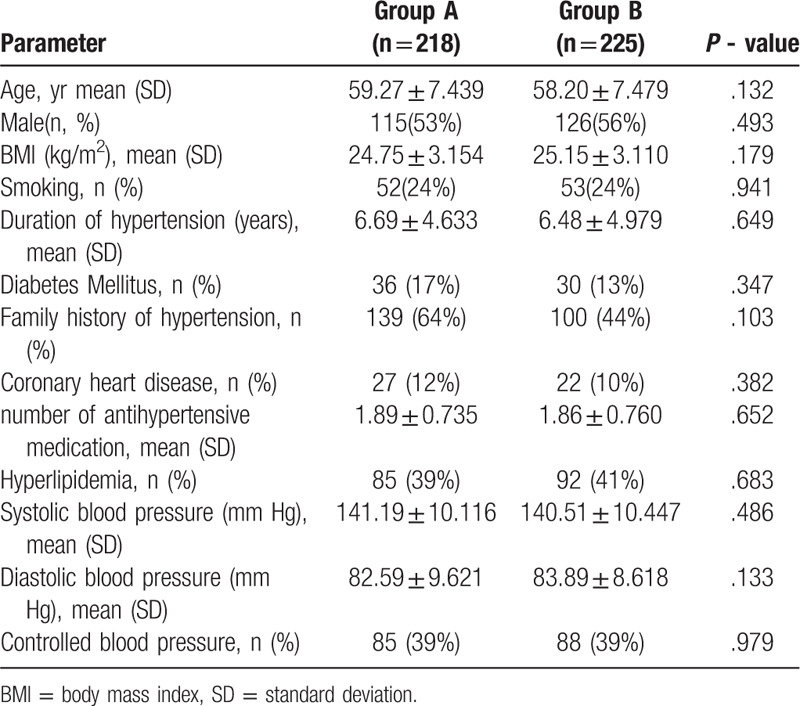
Demographics and baseline characteristics of participants (n = 443).

### 3.2 Primary outcomes

At 6th month, The mean SBP and DBP were significantly lower than that at the start in both groups. Significant differences were observed in the mean decreases of SBP between the intervention group (-8.99 ± 6.415mm Hg) and the control group (-5.92 ± 6.945 mm Hg), *P* = .00. Significant differences were also observed in the mean decreases of DBP between the intervention group (-7.04 ± 6.135mm Hg) and the control group (-4.14 ± 8.213 mm Hg, *P* = .00, Table 5).

**Table 5:**
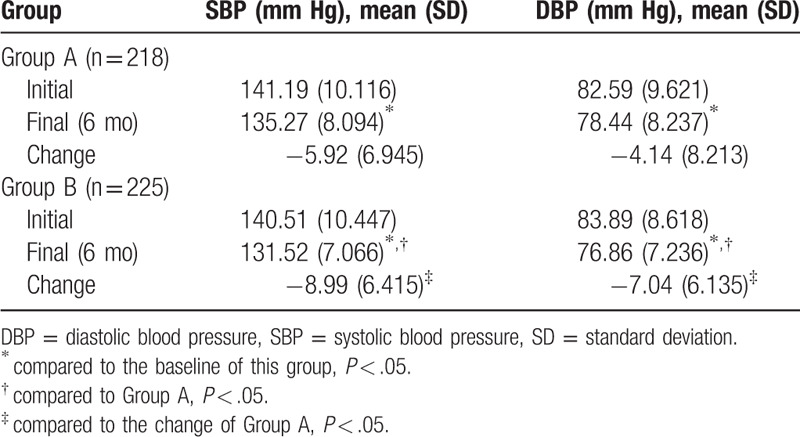
SBP and DBP results for the 2 groups (mmHg, 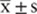).

In total, 85 participants’ blood pressure was categorized as controlled at the start compared to 145 at the end in the control group. In the intervention group, 88 participants’ blood pressure was categorized as controlled at the start to 174 at the end. Both groups had a greater percentage of participants with controlled blood pressure at the end than the start (*P* = .00) (Table 6). The intervention group had a greater percentage of participants with controlled blood pressure than the control group at the end (*P* = .011) (Table 7).

**Table 6:**
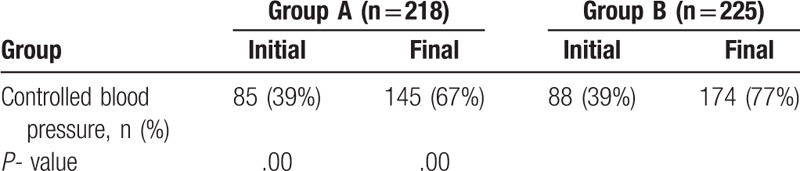
The percentage of participants with controlled blood pressure in the 2 group.

**Table 7:**
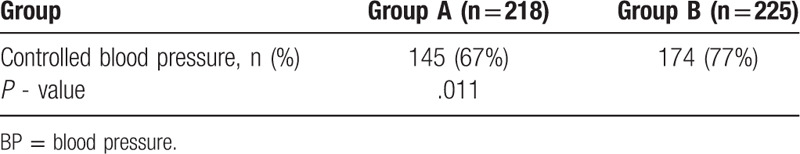
The comparison of the percentage of participants with controlled BP at the end.

### 3.3 Secondary outcomes

At 6th month, The medication adherence were both improved in the 2 groups(Table 8). 8 participants in the intervention group were categorized as high adherence, 94 categorized as medium adherence, and 123 categorized as low adherence compared to the 4 categorized as high adherence, 66 as medium adherence and 148 as low adherence in the control group. the intervention group had higher medication adherence than the control group (Table 9).

**Table 8:**
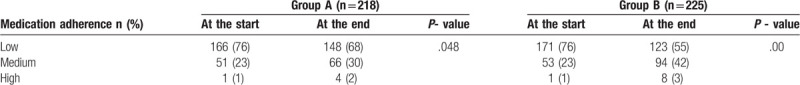
The medication adherence of the 2 groups.

**Table 9:**
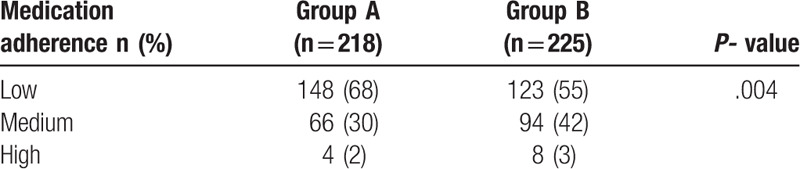
The comparison of medication adherence of the 2 groups at the end.

## 4 Discussion

Hypertension is a growing public health problem worldwide, the rate of blood pressure control is still unsatisfactory. Many m-health applications are applied to the management of hypertension, The developers claim apps can improve the medication adherence and control the blood pressure. Some researches show that there may be as many as 1.7 billion mhealth users globally by 2018.^[3]^ But few m-health apps have been tested in clinical trials, so the effectiveness of the apps in hypertension lacks reliable datas. A systematic review in 2012 analyzed 147 m-health apps which targeted medication adherence, the results showed that there were no effective data to prove that these apps could improve medication adherence.^[5]^ Although there had some studies exploring the effectiveness of apps. Kim et al^[6]^ evaluated the use of a wireless self-monitoring program which had functions of reminders for monitoring, information about the disease condition, and general health behavior recommendations, results showed that the wireless self-monitoring program could improve patients’ behaviors and blood control, but no differences were observed with respect to medication adherence. MedISAFE-BP trial is a randomized trial for Americans to evaluate the effect of m-health apps. Morawski et al found that MedISAFE-BP app could help blood pressure control and improve medication adherence, but the follow-up period was only 12 weeks.^[7]^ Jha et al^[8]^ carried out a multicenter,cluster randomised,12-month, controlled trial to expolre the effectiveness of mWellcare in India, the conclusion was that mWellcare was effective for blood pressure control. Also, a clinical trial in Iran showed that BPMAP-APP was good for blood pressure control and medication adherence.^[9]^ Above all, we could know that there were not enough datas to prove the effectiveness of apps for hypertension, also some clinical trials had different views. Trials about Chinese was little, we know that there are different characteristics and race diversities between Chinese and other countries, so we can’t extend the conclusion that apps are effective for blood pressure control and medication adherence to Chinese directly. Our trial was a multicenter, randomized, controlled study for Chinese. In this trial, participants with primary hypertension who used the “Yan Fu” app had significantly greater reductions in both SBP and DBP than participants in the control group. The intervention group also had a higher percentage of participants with controlled blood pressure than the control group, which meant that the “Yan Fu” app was effective for hypertension management. Also, from the results we could conclude that the use of the app can improve medication adherence. We analyzed that the app could help control blood pressure mainly through improving medication adherence. Actually 1 of the reasons why the rate of blood pressure control is low can be attributed to low medication adherence. Maybe the reason of medication nonadherence can be divided into unintentional or intentional, though the cause of medication nonadherence varies among patients. Unintentional nonadherence involves failing to do for some reasons such as forgetting.^[5]^ The “YanFu” app have the functions of reminder, monitoring blood pressure,counseling,education so that users could remember to take medicine in time and pay more attention to the disease. In our trial the participants monitored blood pressure with home blood pressure monitors, which could minimize the “white-coat effect” of artificially elevated blood pressure in clinic compared to previous hypertension trials. Also, Studies have shown that a 5 mm Hg reduction in SBP could cause clinically meaningful decreases in the incidence of stroke and coronary heart diseases.^[10]^ So maybe we could extend the follow-up period to explore whether apps can reduce the disability rate and mortality of hypertension.

## 5 Limitations

There were several limitations:

(1) It was difficult to observe the effect of the smartphone application on long-term outcomes such as major adverse cardiac and cerebrovascular events and lead to discrepancies in results because of the follow-up period was too short;
(2) the measurement of medication adherence was too subjective which may impact the reliability of the results.^[11]^

## 6 Conclusion

The use of m-Health apps could help to reduce blood pressure and improve blood pressure control and medication adherence, which can play an important role in reducing the economic and social burden of hypertension in humans. In the future, we may promote the use of m-Health applications to manage hypertension and even other chronic diseases.

## Acknowledgments

The application and the sphygmomanometers was provided by Shanghai O2O Care Company for free. We thank Xuejun Wang who is the president of the company, the APP study group which was constituted by 38 hospitals in Chongqing and the patients. This research did not receive any specific grant from funding agencies in the public, commercial, or not-for-profit sectors.

## Author contributions

**Article drafting:** Ke Gong, Yu-Ling Yan, Qiang She.

**Collaborators:** Zhong-lin Xu, Guang-yan Mei, Hui Zhang, Yin-pin Zhou, Chun-hui Hou, Ting-rong Liu, Hong-dong Xia, Fang-qiong Li, Yong-yue Tian, Xue-mei Liao, Ze-hui Ao, Xiao-hua Pang, Wei Zhang, Xiao-ke Wang, Song-tao Zhang, Zhen-yun Wu, Dong Ma, Qiang Li, Chun-lin Xiong, Yue-long Yuan, Jun-lan Li, Qing-bin Liao, Shi-quan Fu, Ming-chen Li, Chang-qing Yu, Wen-li Wu, Chun Yang, Jun Chen, Jing Chen, He-ping Qin, Hong-bin Cheng, Heng-hua Dai, Sha-sha Zeng, Hong-jun Liu, Feng Yan, Qing Zhang, Fang He.

**Data collection and analysis:** Ke Gong, Yu-Ling Yan, Yue Han, Ya Zou, Xin-yu Zou, Hong Huang, APP Study Group, Qiang She.

**Study design:** Ke Gong, Yu Li, Jun Du, Jing Wang, Qiang She.
